# Assistive Navigation Using Deep Reinforcement Learning Guiding Robot With UWB/Voice Beacons and Semantic Feedbacks for Blind and Visually Impaired People

**DOI:** 10.3389/frobt.2021.654132

**Published:** 2021-06-22

**Authors:** Chen-Lung Lu, Zi-Yan Liu, Jui-Te Huang, Ching-I Huang, Bo-Hui Wang, Yi Chen, Nien-Hsin Wu, Hsueh-Cheng Wang, Laura Giarré, Pei-Yi Kuo

**Affiliations:** ^1^ Department of Electrical and Computer Engineering, Institute of Electrical and Control Engineering, National Chiao Tung University, Hsinchu, Taiwan; ^2^ Department of Electrical and Computer Engineering, Institute of Electrical and Control Engineering, National Yang Ming Chiao Tung University, Hsinchu, Taiwan; ^3^ College of Technology Management, Institute of Service Science, National Tsing Hua University, Hsinchu, Taiwan; ^4^ Department of Engineering, University of Modena and Reggio Emilia, Modena, Italy

**Keywords:** UWB beacon, navigation, blind and visually impaired, guiding robot, verbal instruction, indoor navigation, deep reinforcement learning

## Abstract

Facilitating navigation in pedestrian environments is critical for enabling people who are blind and visually impaired (BVI) to achieve independent mobility. A deep reinforcement learning (DRL)–based assistive guiding robot with ultrawide-bandwidth (UWB) beacons that can navigate through routes with designated waypoints was designed in this study. Typically, a simultaneous localization and mapping (SLAM) framework is used to estimate the robot pose and navigational goal; however, SLAM frameworks are vulnerable in certain dynamic environments. The proposed navigation method is a learning approach based on state-of-the-art DRL and can effectively avoid obstacles. When used with UWB beacons, the proposed strategy is suitable for environments with dynamic pedestrians. We also designed a handle device with an audio interface that enables BVI users to interact with the guiding robot through intuitive feedback. The UWB beacons were installed with an audio interface to obtain environmental information. The on-handle and on-beacon verbal feedback provides points of interests and turn-by-turn information to BVI users. BVI users were recruited in this study to conduct navigation tasks in different scenarios. A route was designed in a simulated ward to represent daily activities. In real-world situations, SLAM-based state estimation might be affected by dynamic obstacles, and the visual-based trail may suffer from occlusions from pedestrians or other obstacles. The proposed system successfully navigated through environments with dynamic pedestrians, in which systems based on existing SLAM algorithms have failed.

## 1 Introduction

According to the World Health Organization, the number of people worldwide who are blind and visually impaired (BVI) is 286 million; therefore, the development of suitable navigation aids is essential. Currently, due to their low cost and reliability, white canes are the most commonly used navigation aid by BVI people (Tapu et al., 2014). Guide dogs are another navigation aid for helping BVI people avoid contact with other pedestrians; guide dogs also provide companionship. However, according to the International Guide Dog Federation, guide dogs are unavailable in many regions to most BVI people who require them due to the high cost of animal training and determining suitable dog–user pairings. Furthermore, some environments are not service animal friendly. Finally, according to the federation, it breeding, raising, training, and placing a guide dog costs approximately US *$*50,000. This cost may be unaffordable for many BVI people. Over the past 2 decades, numerous efforts have been made to develop navigation robots for assisting BVI people. Several types of robotic navigation aids have been developed, including robotic canes (Ulrich and Borenstein, 2001; Wang et al., 2017), walkers (Wachaja et al., 2015, 2017), and suitcases (Kayukawa et al., 2019, 2020), as well as other mobile platforms (Kulyukin et al., 2006).

**FIGURE 1:**
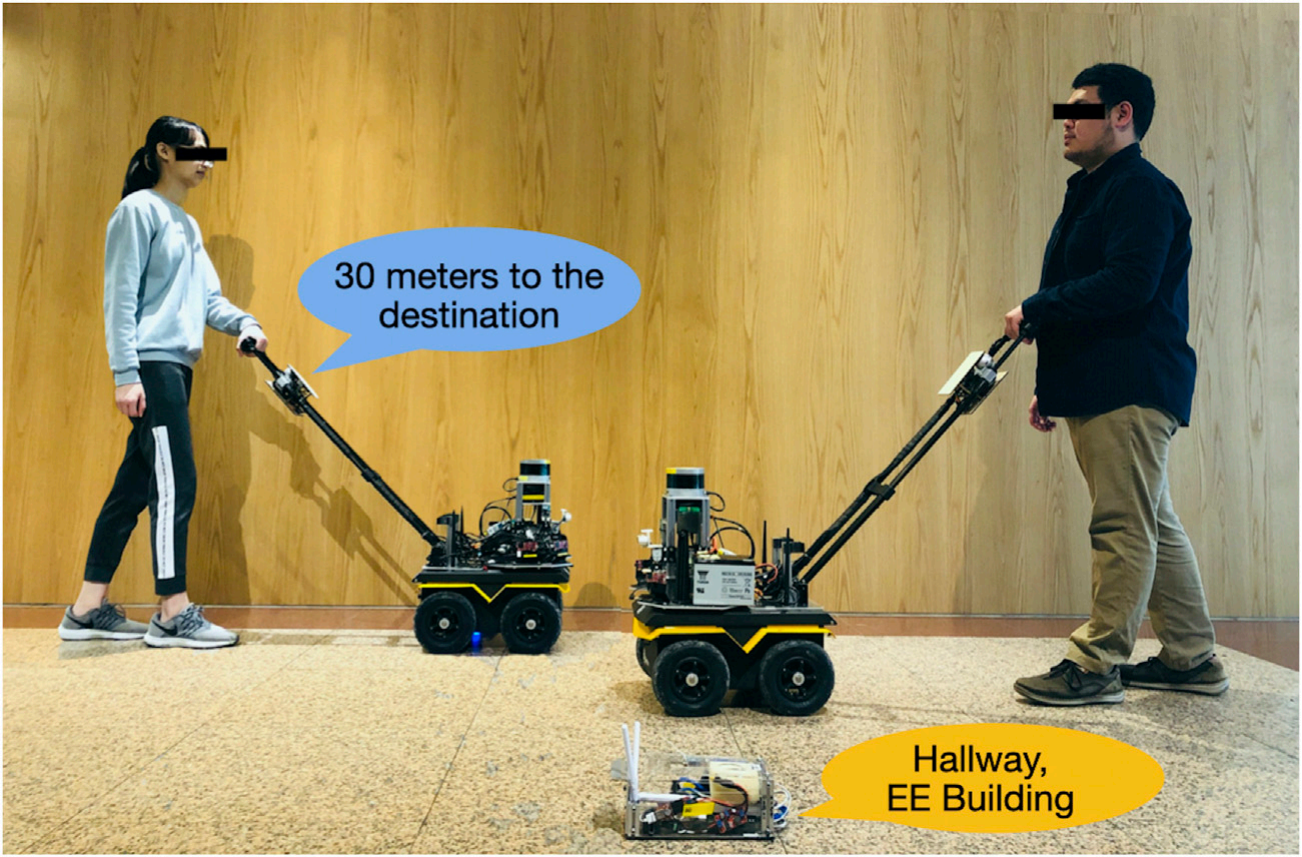
Proposed navigation solution for BVI people. The designed system includes a guiding robot, an interactive harness, and UWB beacons. The system provides on-harness or on-beacon verbal semantic feedback. Usability studies were conducted to ensure that the designed guiding robot is easily accessible to BVI users.

Guiding robots execute navigation, localization, and obstacle avoidance tasks by sensing the surrounding environment (Figure 1). Ulrich and Borenstein (2001) proposed the “GuideCane” robot, which is equipped with a white cane, for navigation assistance. When the ultrasonic sensors in the GuideCane robot detect an obstacle, the embedded computer determines a suitable direction of motion, and steers the robot (and user) around the obstacle. Kulyukin et al. (2006) designed a wheeled mobile platform by using a laser rangefinder and radio-frequency identification (RFID) sensors to navigate indoor environments with preinstalled RFID tags. Gharpure and Kulyukin (2008) proposed a robot to help blind people shop. This robot guides users in a store and informs them of item prices. Li and Hollis (2019) used a human-sized mobile robot and human interaction module to provide active navigation guidance similar to that provided by humans to BVI users. Nagarajan et al. (2014) developed a human-following system based on a single two-dimensional (2D) LiDAR mounted on the pan–tilt turret of a ballbot. Kayukawa et al. (2019) proposed a suitcase guiding system named BBeep, which includes a sonic collision warning system for predicting the motions of nearby pedestrians. An active sound is emitted to alert pedestrians and the blind user to reduce the risk of collisions. However, this system is more suitable for use in crowded environments, such as airports, than in quiet places, such as hospitals, libraries, and restaurants. Chuang et al. (2018) adopted a learning-based approach for enabling BVI people to navigate pedestrian environments by trails-following of the current tactile guide paths. Kayukawa et al. (2020) developed a guiding suitcase with two RGB-depth (RGB-D) cameras and employed a convolutional neural network (CNN)-based object detector for identifying pedestrians. A LiDAR-based simultaneous localization and mapping (SLAM) algorithm was used to estimate the egomotion and planned path. When pedestrians walk across the planned path, the blind user receives an alert from the system to adjust their walking speed or stop without changing their path (the on-path mode). If the planned path is blocked, the system recommends an alternative path around the pedestrians (the off-path mode). Unlike the BBeep system, the system of Kayukawa et al. (2019) avoids active sound emission and the creation of unnecessary attention in quiet environments.

There have been significant developments in two major techniques on simultaneous localization and mapping (SLAM) as well as motion planning, which enable robotic systems to navigate in GPS-denied indoor environments. Nevertheless, SLAM systems tend to have difficulties with dynamic environments or textureless scenes, and are vulnerable to perception outliers (Lajoie et al., 2019b). The unrecoverable errors from perception may make it infeasible for safe motion planning. To achieve accurate blind navigation, the degradation of SLAM algorithms in dynamic environments must be considered. Although many studies have been conducted on the detection and avoidance of static obstacles, to our knowledge studies on collision avoidance with dynamic pedestrians for blind navigation are scarce. In contrast to the classic map-localize-plan approaches, deep reinforcement learning (DRL) realizes goal navigation and collision avoidance capabilities, where robots learn to navigate through trial and error without building a mapping.

For localization to provide assistance in blind navigation, a common approach is by the use of a beacon network, which contains beacons that are predeployed in the environment. Such deployment of beacons showed localization accuracy within approximately 1.65 m on average by detecting the Bluetooth signals from a beacon Sato et al. (2017). Guerreiro et al. (2019) further designed an independent mobility system for visually impaired travelers in an airport, including four routes of potential scenarios for independent navigation. The aforementioned system helped the participants reach their destination successfully; however, although the participants provided positive feedback overall, the system failed in tasks such as navigating door entrances due to the meter-scale position error.

Voice navigation (e.g., through smartphones) is commonly used for blind navigation. Sato et al. (2017) provided turn-by-turn instructions as well as information on landmarks and nearby points of interest (POIs). The assessment results obtained for the aforementioned application by Guerreiro et al. (2019) with more than 14 participants indicated its feasibility in the real world. The subjective responses of the participants confirmed that semantic features are helpful for constructing spatial maps for navigation. Furthermore, most participants held a positive attitude toward navigating independently in any unfamiliar environment at different times of the day. However, voice navigation requires users to follow frequent navigational cues carefully. Therefore, navigation by using mobile robots is preferred to voice navigation for reducing the cognitive loads of users.

This study is an extension of our previous research (Wang et al., 2017; Chuang et al., 2018; Lu et al., 2021). The contributions of this study are as follows:

1. We propose a mobility aid that extends the navigation assistance provided by a white cane and guide dog with high reliability. The physical interface was constructed using a rigid handle equipped on the proposed guiding robot. The body yaw produces a haptic signal to allow the user to follow its movement intuitively.
2. A DRL approach is presented for a robot designed to follow selected waypoints and avoid static or dynamic obstacles. To achieve more precise positioning than existing Bluetooth Low Energy (BLE) solutions, an ultrawide-bandwidth (UWB) localization system is proposed to overcome the influences of dynamic obstacles in the environment, which have been found challenging by classic map-localize-plan approaches.
3. An integrated UWB/Voice Beacon is designed to form a beacon network and to provide voice feedback. Voice instructions is provided through on-beacon and on-robot feedback, such as travel destination information, and responds to queries for physical assistance, such as whether to go, stop, or change speed when traveling. An investigation was conducted with BVI users to evaluate the performance of the proposed system.

## 2 Related Work

### 2.1 Mobile Robot for Assistive Technology

Due to advances in autonomous driving and robotics, the applications of mobile robots have increased considerably. The applications of mobile robots in assistive technology include navigation robots for BVI people and robotic wheel chairs (Hersh, 2015). Wachaja et al. (2015) developed a smart walker for blind people with walking disabilities. This walker consists of two laser range scanners for sensing local obstacles and a standard notebook computer to perform all computations for reaching the desired location. Wachaja et al. (2017) modeled human motion with a walker on the basis of recorded trajectories to design a controller that considers the user’s reaction time, rotational radius, and speed.

Similarly, the comfort level is a crucial factor related to robotic wheel chairs. Gulati et al. (2009) formulated discomfort as a weighted sum of the total travel time and time integrals of various kinematic quantities to plan trajectories that minimize discomfort. Morales et al. (2013) presented a “comfort map” that integrates the human factor into the SLAM framework. Subsequently, Morales et al. (2014, 2015) investigated the comfort level through visibility analysis to plan trajectories that minimize the discomfort level.

In contrast to wheelchair users, BVI people tend to use navigation assistance technologies when traveling alone. Users of robotic wheel chairs are often accompanied by a caregiver or companion. Therefore, the design goals of robotic wheel chairs may be different from guiding robots in order to move alongside humans smoothly. Recent work (Kobayashi et al., 2009, 2011, 2011) investigated the interaction between a caregiver and a robotic wheel chair. The robotic wheelchair used an omnidirectional camera and a laser rangefinder (i.e., a 2D LiDAR) to track companions that might be alongside or in front of the wheelchair according to the visual laser tracking technique.

### 2.2 Wearable/Smartphone-Based Navigation Aids—Beacon Network and Voice Feedback

A wearable or smartphone-based device has several advantages, such as small size and light weight. Wearable technologies can be used for navigation aid; however, wearable devices, which are customized and dedicated devices, are sometimes impractical to use without mass production. A smartphone-based indoor assistant application does not require an additional device. This application can provide speech-based inputs, vibrations, sound events, or verbal instructions without the installation of any sensors.

Smartphone-based navigation aids have been used to locate the positions of users’ smartphone using Bluetooth Low Energy (BLE) (Sato et al., 2017). BLE uses 40 channels in the 2.4-GHz band. In general a positioning error of 2.6 m is achieved 95% of the time for a dense BLE network (one beacon per 30 m^2^) (Faragher and Harle, 2015). UWB modules estimate the position of a UWB tag within the space spanned by three or more UWB beacons through triangulation. There have been robotics applications combining onboard UWB tag with inertial measurement units (IMU) or gyroscope sensors, to be localized in environments with pre-deployed UWB beacons (Mueller et al., 2015; Mai et al., 2018; Cao et al., 2020). Nevertheless, to our knowledge the uses of UWB modules and DRL methods have not been reported in the literature for navigation aids to BVI users.

### 2.3 Nonvisual Interfaces

All the aforementioned guiding robots and some wearable/smartphone-based navigation aids are equipped with nonvisual and haptic feedback instead of auditory signals. Audio feedback is considered undesirable and restrictive in noisy environments and interferes with the perception of ambient sound for situational awareness. Wachaja et al. (2017) paired the walker with a wirelessly connected vibrating belt that has five motors. This belt must be worn by the user around the waist, and two additional vibration motors were attached to the walker handles. The vibration intensity and pattern of each motor represent navigation commands or the distance to the closest obstacle within the available angular range. Users were found to favor the vibration motors in the handles over those in the belt. The belt provided a higher spatial resolution than the handles did and thus conveyed more information. Wang et al. (2017) developed a system that provides feedback to the user through a haptic device with five vibration motors on a belt. This belt vibrates at a suitable location on the user’s body or through a braille display, which allows the user to feel the occupancy grid or object description with their fingers. These interfaces reflect the local state of the environment to assist the user in navigating objects, such as empty chairs. Users reported that they could easily acclimatize to the haptic belt interface and found it moderately comfortable. Moreover, the signals were readily interpretable. Braille displays offer rich high-level feedback in a discrete manner; however, the reaction times of users are long when using such displays because the sweeping of fingers on braille cells requires time. Katzschmann et al. (2018) proposed the embedding of small and lightweight sensors with haptic devices into clothes for enabling discrete environment navigation. Such an embedded system, which includes a haptic feedback array, allows users to sense low- and high-hanging obstacles as well as physical boundaries in their immediate environment for local navigation. The aforementioned system allows blind people to complete key activities that they would normally perform with a white cane in a similar amount of time. Kayukawa et al. (2020) introduced tactile shape-changing interfaces to indicate fine directions. The “Animotus” feedback system is a cube-shaped interface that conveys heading directions, and it allows users to adjust the status of the robot. Nagarajan et al. (2014) combined coarse interactive forces with speech cues to fine-tune the position of the person being led. Other solutions have been proposed for safe navigation with wearable and nonvisual feedback devices, refer to (Wang et al., 2017).

## 3 Hardware and Feedback System of the Proposed Guiding Robot

### 3.1 Guiding Robot Hardware

**FIGURE 2:**
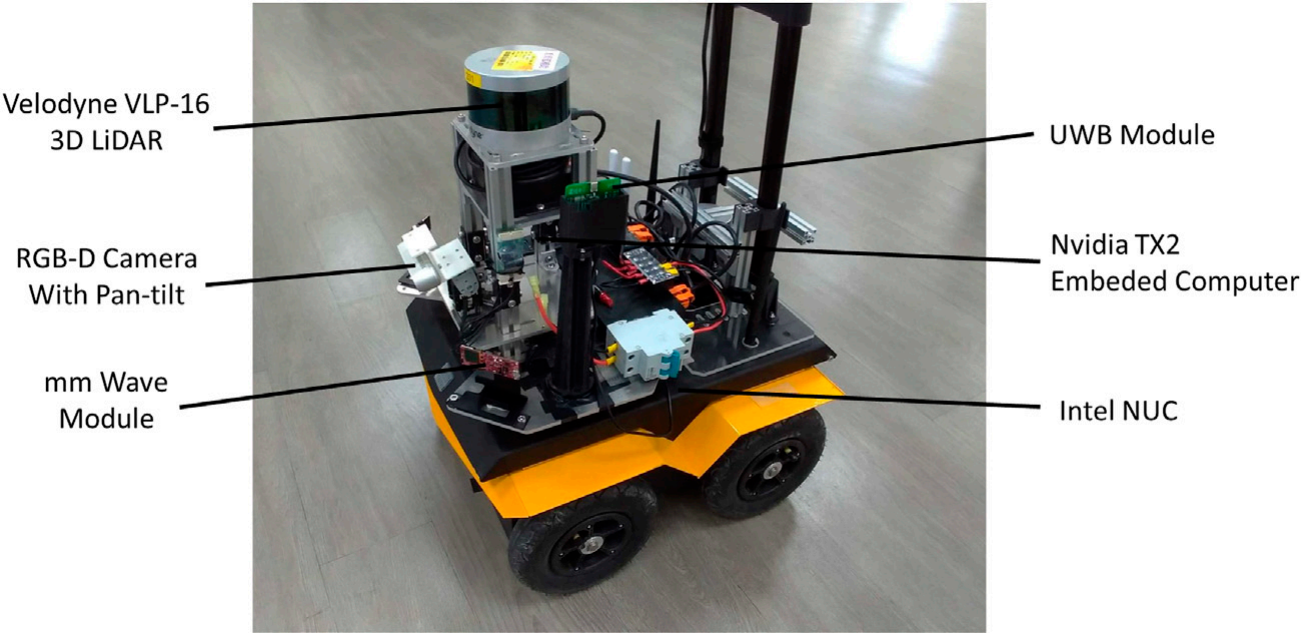
Proposed guiding robot system.

The proposed guiding robot was constructed on the Clearpath Jackal UGV, which is suitable for indoor and outdoor terrains (Figure 2). In this study, the proposed robot was navigated mainly on flat surfaces and a few stairs, which represent most urban environments. The robot contains a sensor tower for mounting all the sensors, including a Velodyne LiDAR VLP16 sensor (on top of the robot), a RealSense D435 depth camera with a pan–tilt motor, and an mmWave module. The mounted sensors are used by the perception system for tasks such as obstacle detection and wall following. The computation units include an Intel NUC computer connected to direct current motors with pulse-width modulation control and an NVIDIA Jetson TX2 embedded system with 8 GB of graphics processing unit/central processing unit memory for onboard processing. Most of the computations, including those for SLAM that require considerable computation resources, were executed on an Intel computer. The DRL computations were the only calculations that required parallel computing; therefore, these computations were executed in the Jetson TX2 embedded system. The proposed robot also includes a Pozyx UWB module and UniFi WiFi access point. The dimensions of the robot are 51 × 43 × 25 cm^3^ (length × width × height). The robot system, including the sensors, computation units, and batteries, weighs approximately 25 kg; thus, the proposed system can be suitably maneuvered by users. An interactive handle is mounted to provide guiding signals and semantic information. The overall cost of the robot is approximately USD 17,000, which is three times cheaper than the cost of a guide dog.

### 3.2 Voice Feedback System on the Handle and Beacon

**FIGURE 3:**
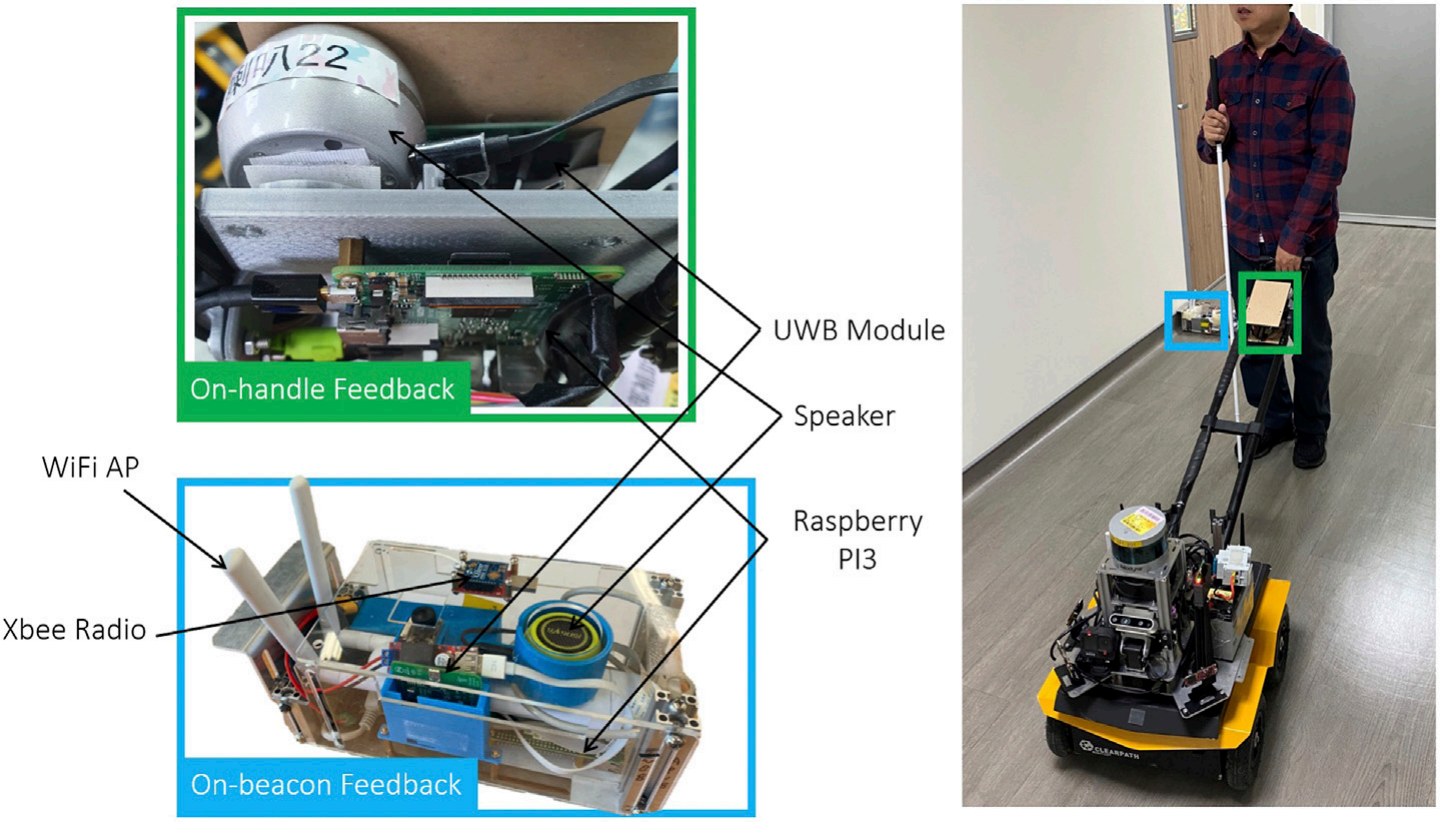
The integrated Ultrawide-Bandwidth (UWB)/Voice beacon contains a Raspberry Pi (RPi) embedded computer, a battery, a UWB module and a speaker for verbal feedback. Communication devices include a WiFi Access Point (AP), XBee radio, and a UWB module.

The UWB/voice beacons used in this study (Figure 3) were designed to be self-sustaining, including a Raspberry Pi embedded computer, UWB module, communication modules, a battery, and speaker for verbal feedback. The UWB module provides ranges between other UWB modules at the robot and other nearby beacons for the subsequent robot state estimation. The Raspberry Pi computer regulates all the main functions of the beacon, including the communication of the UWB modules and XBee module, the verbal feedback, and speaker control. The communication devices include a WiFi access point, an XBee radio, and a UWB module. The WiFi access point mesh network enables real-time monitoring and data logging of the ground truth for safety purposes. The low-bandwidth but stable XBee radio serves as an emergency stop if necessary. In addition to range measurements, the UWB module requests verbal feedback.

Semantic feedback is provided through on-handle or on-beacon verbal notifications for different purposes. The on-beacon verbal notifications are designed to provide situational awareness regarding not only the target but also the POIs in the surroundings, such as room numbers, stores, water fountains, or toilets. The speaker allows BVI people to construct a mental map of the environment according to nearby POIs.

The main purpose of the on-handle verbal feedback is to provide individual pieces of information, including the robot status and trip status, so that users can intuitively understand current circumstances. Therefore, the on-handle feedback provided in this study included an update regarding the distance remaining whenever a POI was reached. A POI was reached approximately every 10–20 m.

The interactive handle used in this study (Figure 3) is similar to that used in the study of Chuang et al. (2018). A sliding button is added to the handle to allow users to adjust the robot’s velocity among seven levels. A push button is used to stop the robot in an emergency. The on-handle device is similar to the proposed UWB/voice beacon, including a speaker, UWB module, and WiFi module that communicates with the guiding robot. The on-handle voice feedback is designed to provide high-level navigation cues that cannot be provided by a white cane or guide dog, such as notifying the user of an intersection.

## 4 The Proposed Navigation Method

Autonomous navigation is a challenge, especially in dynamic environments that are often cohabited by various mobile agents. DRL models (Everett et al., 2018) have been developed to explore navigation policy through an indoor social environment. These algorithms predict an optimal path by using a value network trained through DRL. They use the positions and velocities of a robot and surrounding agents (e.g., pedestrians) as the input. Chen et al. (2017) further encodes social norms in the reward function to enable the trained robots follow socially aware behaviors. In this study, instead of predicting an optimal path, the DRL agent directly selected an optimal control command with respect to the state.

We carried out two proposed navigation methods using 1) DRL and UWB localization, and 2) SLAM and DRL, as well as a baseline using SLAM and A* planning. All methods consider the comfort level by either a action soft update (for DRL) or a pure pursuit controller (for A*) while the robot tracked the path and computed the required velocity and angular velocity. We carried out a velocity smoother as a bounded linear function with the maximum and minimum acceleration as the upper bound and lower bound to avoid sudden acceleration and abruptly stop.

### 4.1 Proposed 1: Navigation Using Deep Reinforcement Learning and Ultrawide-Bandwidth Localization

**FIGURE 4:**
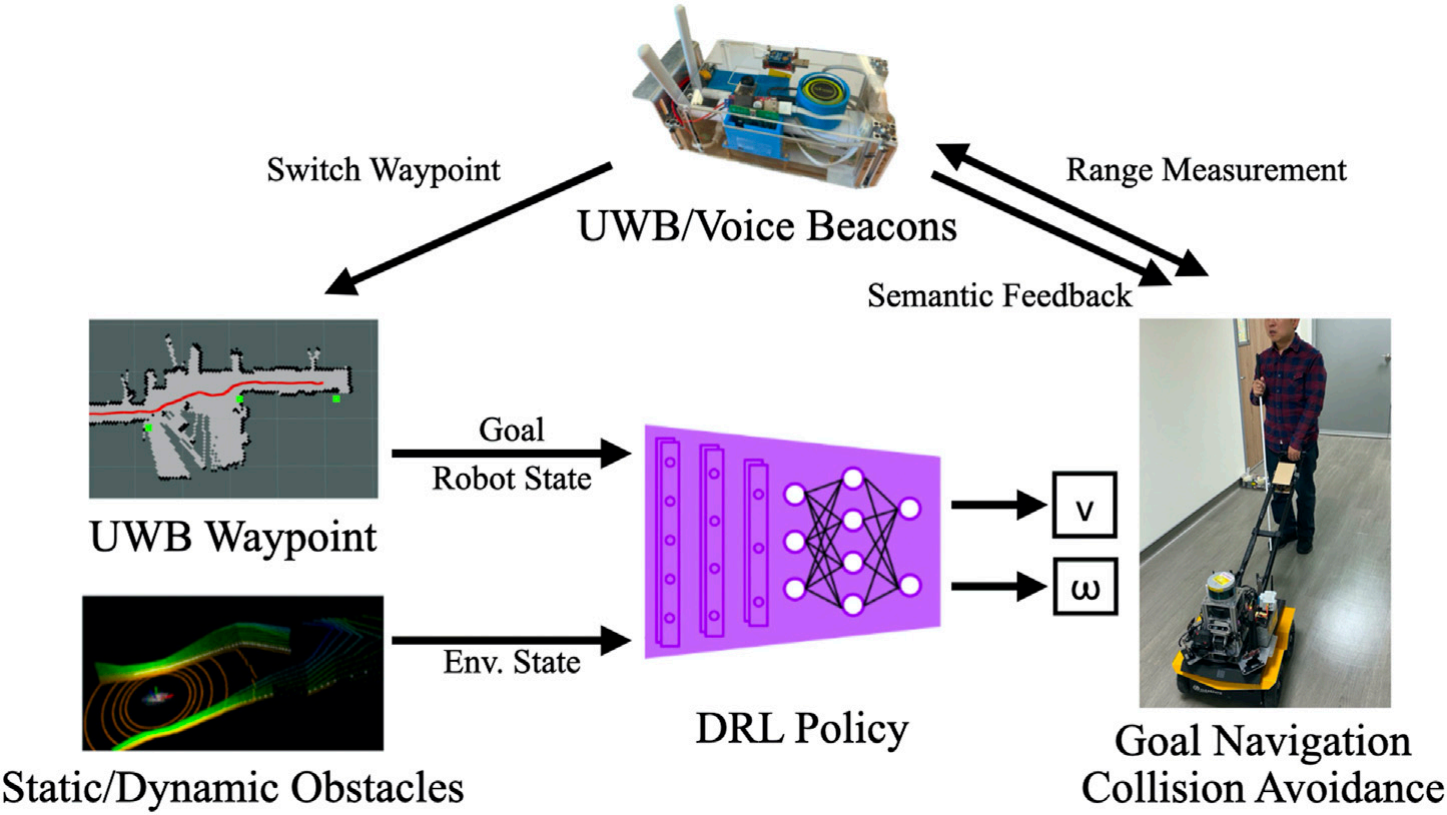
Key components of the proposed system. The Deep Reinforcement Learning (DRL) algorithm obtains goal information from a series of designated waypoints through Simultaneous Localization and Mapping (SLAM) or UWB localization. Moreover, it obtains environmental information from LiDAR point clouds. The adopted algorithm facilitates the proposed guiding robot to achieve navigation and collision avoidance for BVI users. The UWB/Voice beacons estimate ranges as thresholds for switching waypoints and provide semantic feedback through the handle.

The key components of the designed system are displayed in Figure 4. The UWB/voice beacons are placed at certain POIs, such as restrooms, elevators, or gates and doors, as waypoints and other location to enable positioning. The LiDAR point clouds indicate the state of the environment, and the destination location (the current waypoint) estimated using the SLAM algorithm or through UWB localization is directly fed into the navigation agent. The navigation agent is a DRL policy that outputs a velocity *v* and an angular velocity ω to the motor controller without using a path planning algorithm. Excluding the communication between the robot and the beacons, all the remaining messages were exchanged between the components through the robot operating system (ROS). The pseudocode of this system is presented in Algorithm 1.

**Algorithm 1:** The proposed navigation using DRL and UWB Localization.
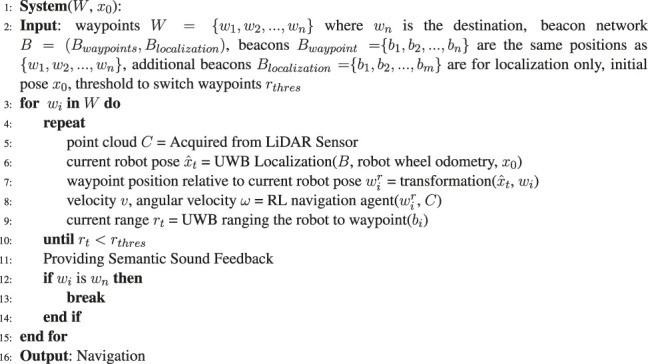

#### 4.1.1 Deep Reinforcement Learning Settings and Background

The sequential decision-making problem can be formulated as a partially observable Markov decision process (POMDP) that can be solved through DRL. POMDP is defined as a six-tuple $\left(S,A,P,R,\rho_{0},\gamma \right)$ where *S* denotes the state space, *A* denotes the action space, P(s'|s,a) denotes the transition probability, *R* denotes the reward function, $\rho_{0}$ denotes the initial state distribution, and *γ* denotes the discount factor. The goal is to optimize deep policy networks by maximizing the cumulative reward. In this study, we defined the state space as the data points from the LiDAR and the goal point relative to the guiding robot. The action space is the normalized linear and angular velocity of the robot.

The reward function is defined and described below. The function of the designed robot is to reach the destination while avoiding collisions. The transition probability is the probability of one agent moving from one state to another state under the condition of an action being executed. In early studies on RL and the Markov decision process, the transition probability was defined by a matrix expressing the probability of moving from one state to another. However, in the real world, countless states exist, and describing these states in matrix form is impossible. In this study, the transition probability was defined by the simulator of the robot. The simulator embedded the transition probability but did not explicitly specify it. The robot then learned the transition probability implicitly and attempted to reach the highest accumulated reward in the training phase. The discount factor determined how the agent cared about the distant and immediate futures. We set the discount factor to 0.99, as suggested by Barth-Maron et al. (2018).

We adopted the DRL method proposed by Lu et al. (2021) to train our agent. In contrast to CADRL, the proposed method directly maps the state space (i.e., the LiDAR points) to control commands. Furthermore, the proposed method allows continuous control, which increases robot flexibility. On train a generalized model, a Gazebo environment was used as the training environment in this study. Gazebo is a widely used simulator in robotics that can accurately and efficiently simulate populations and sensors of robots in complex indoor and outdoor environments. The training environment, which contained tunnels of diverse sizes and shapes, was designed by the Subterranean Challenge team from DARPA. The simulated vehicle had similar properties to those of the Husky UGV manufactured by Clearpath. An DRL agent is trained using the state, action, and reward, which are defined in the following text.

• Observations: We used 240°, from −120° to 120° with a resolution of 1° in the field of view of the LiDAR points. We concatenated four consecutive frames of range data and 10 frames of relative positions to the goal point as a single observation space.
• Actions: The actions were designed as linear and angular actions. Linear actions were limited to [0,1], and angular actions were limited to [−1, 1].
• Reward: To ensure that the robot reaches the goal and avoids collisions simultaneously, we established a dense reward function as follows: 1) relative position toward the goal, 2) reaching the goal, and 3) penalty for collision.

$$r\_goal=\{\begin{array}{ll} 0.2\;if\;\text{Toward the goal} & \\ -0.2\;else & \end{array}$$

$$r\_reach=\{\begin{array}{ll} 100\;if\;\text{Reach the goal} & \\ 0\;else & \end{array}$$

$$r\_collision=\{\begin{array}{ll} -10\;if\;\text{Collision} & \\ 0\;else & \end{array}$$

r=r_goal+r_reach+r_collision

#### 4.1.2 Training

The developed model was trained using the Distributed Distributional Deep Deterministic Policy Gradient (D4PG) Barth-Maron et al. (2018). During the training, the agent extracts information from the actor network to collect data (i.e., the state-action-reward triad) and stores these data in a “replay buffer.” The algorithm then samples from the replay buffer to train the agent. The navigation agent is trained only with simulated data and no real-world data. This method allows the achievement of distributed training, which involves obtaining experience from multiple agents in multiple simulators simultaneously. The aforementioned method has an actor–critic structure to train the agent. Unlike other actor–critic methods, such as the Deep Deterministic Policy Gradient (DDPG) Lillicrap et al. (2019), the D4PG uses a distributional critic network to enable robust value estimation.

#### 4.1.3 Network Architecture

The critic and actor networks share a similar architecture. First, a frame of stacked one-dimensional LiDAR points is fed to a one-dimensional CNN network. The CNN network extracts features for the subsequent network. Then, the flattened features are concatenated with the stacked goal points as a representation of the state. Frames are stacked so that the agent can learn from the inputs for a certain duration instead of for a single timestamp. A fully connected layer serves as the strategy-making network and uses the representation to generate the best action (i.e., *v* and *ω*) for the actor network or the best value estimation for the critic network.

#### 4.1.4 Action Soft Update

After the policy network is trained, the agent can navigate with nearly no collisions. However, the agent may learn an undesired action, switching the direction of the angular velocity frequently, which causes the robot to weave often. This motion may cause unnecessary movement that decrease comfort level for users. To maintain the direction of angular velocity, we applied a soft update technique.

$$\omega_{t}=\lambda \omega_{t}+\left(1-\lambda \right)\omega_{t-1}$$ (1)

where $\omega_{t}$ is the angular velocity at time *t*, $\omega_{t-1}$ is the angular velocity at time t−1 and λ is the update parameter.

#### 4.1.5 Ultrawide-Bandwidth Localization and Extended Kalman Filter Updates

The Pozyx UWB modules provide positioning capability through trilateration. Geometrically, a 2D position can be determined by performing ranging with three reference points whose positions are known. The reference points or modules are referred to as beacons, and the positioning module is referred to as the “tag”.

To perform trilateration, the well-known two-way-range method was used for the tag to perform ranging from the tag to beacons. In the aforementioned method, ranging is performed by sending a packet back and forth from one module to other modules and calculating the packet transmission time. A disadvantage of this method is that it does not scale well to large areas, where more than four beacons are required. A module might perform ranging to an unachievable beacon or an excessive number of beacons, which is time intensive. The provided library optimizes the aforementioned method by tracking the position of the tag and only performing ranging with the closest beacons.

The Pozyx modules perform positioning similar to the global positioning system through UWB beacons. However, navigation cannot be conducted through positioning alone; the pose (i.e., position and orientation) of the robot must also be determined. Therefore, an extended Kalman filter (EKF) was used with the aforementioned modules as measurement and the robot’s internal sensor as the control input. The theory of localization with EKF was introduced by Thrun et al. (2005). Sakai et al. (2018) used a simulated global positioning system as the measurement, simulated an internal measurement unit as the control input, and applied EKF for localization. Wheel odometry was used to obtain the control input in the current study; however, an inertial measurement unit sensor can also be used to obtain the control input.

### 4.2 Proposed 2: Navigation With Simultaneous Localization and Mapping and Deep Reinforcement Learning

Beside using UWB localization and EKF as the localization source, the DRL agent can also use SLAM framework as the localization source. We used GMapping (Grisetti et al., 2007) in this work which uses laser scan (2D point cloud) and wheel odometry as the input of the framework. The range measurements of the UWB beacons were used as the hint for waypoint switching. When the range measured by a UWB/Voice beacon is lower than a threshold, which implies that the waypoints have been passed, the system switches to the next waypoint and the UWB/Voice beacon and handle provided verbal feedback to convey environmental information.

**Algorithm 2:** Navigation with SLAM and DRL.
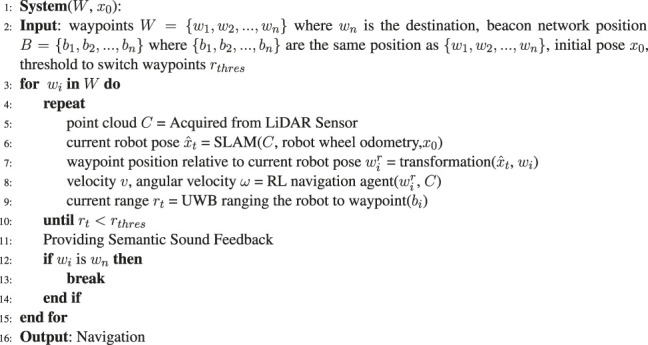

**Algorithm 3:** Classic map-localize-plan approach.
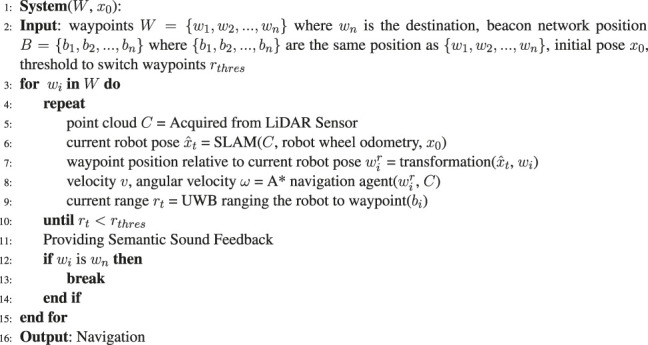

### 4.3 Baseline: Navigation With Classic Map-Localize-Plan (Simultaneous Localization and Mapping and A* Planning)

In addition to the learning-based method, we implemented a classic map-localize-plan method for baseline comparison. We used the A* path planning algorithm of Hart et al. (1968) to determine the optimal (i.e., shortest) path for the robot. The designed system first projects the local LiDAR map to a 2D grid map. After the goal point is provided, the A* path planning algorithm attempts to find the shortest path from the robot to the goal by minimizing the cost (i.e., the distance) in a heuristic manner. We set the grid size to 0.3 m to ensure a sufficient margin between the robot and the obstacles when the robot navigated through narrow passages. Given the planned path from the current robot pose to the goal, i.e., the next waypoint, a pure pursuit controller was used to track the path and computed the desired velocity and angular velocity for the robot. The controller first found the nearest point on the path using a designated look ahead distance. Since the robot navigate in a relatively slow speed, the look-ahead distance was carefully tuned and fixed so that the robot will not overshoot and oscillate along the path too much and cause uncomfortable pivoting behavior. As the nearest point was found, the error of distance and angle of the point relative to the current pose can be derived. The velocity and the angular velocity can therefore been derived.

## 5 Experiment of Navigation in a Pedestrian Environment

**FIGURE 5:**
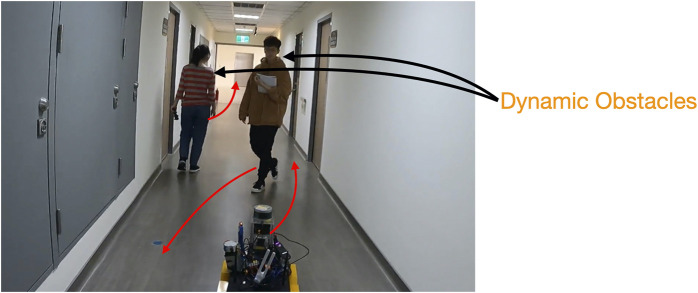
Two people walking in the environment during the experiment for modeling two types of dynamic obstacles. One person traveled in the same direction as the robot. Another person passed the robot numerous times. The robot’s route in the figure is the same as that used in the user study; however, the number of UWB beacons was increased to perform accurate positioning.

The navigation experiment was designed and conducted to examine the effect of the proposed DRL with UWB localization, compared to the two baselines. The SLAM algorithm has been known to perform well in static environments but might fail in environments with numerous pedestrians or occlusions created by dynamic obstacles (Figure 5).

### 5.1 Experiment Setup

**FIGURE 6:**
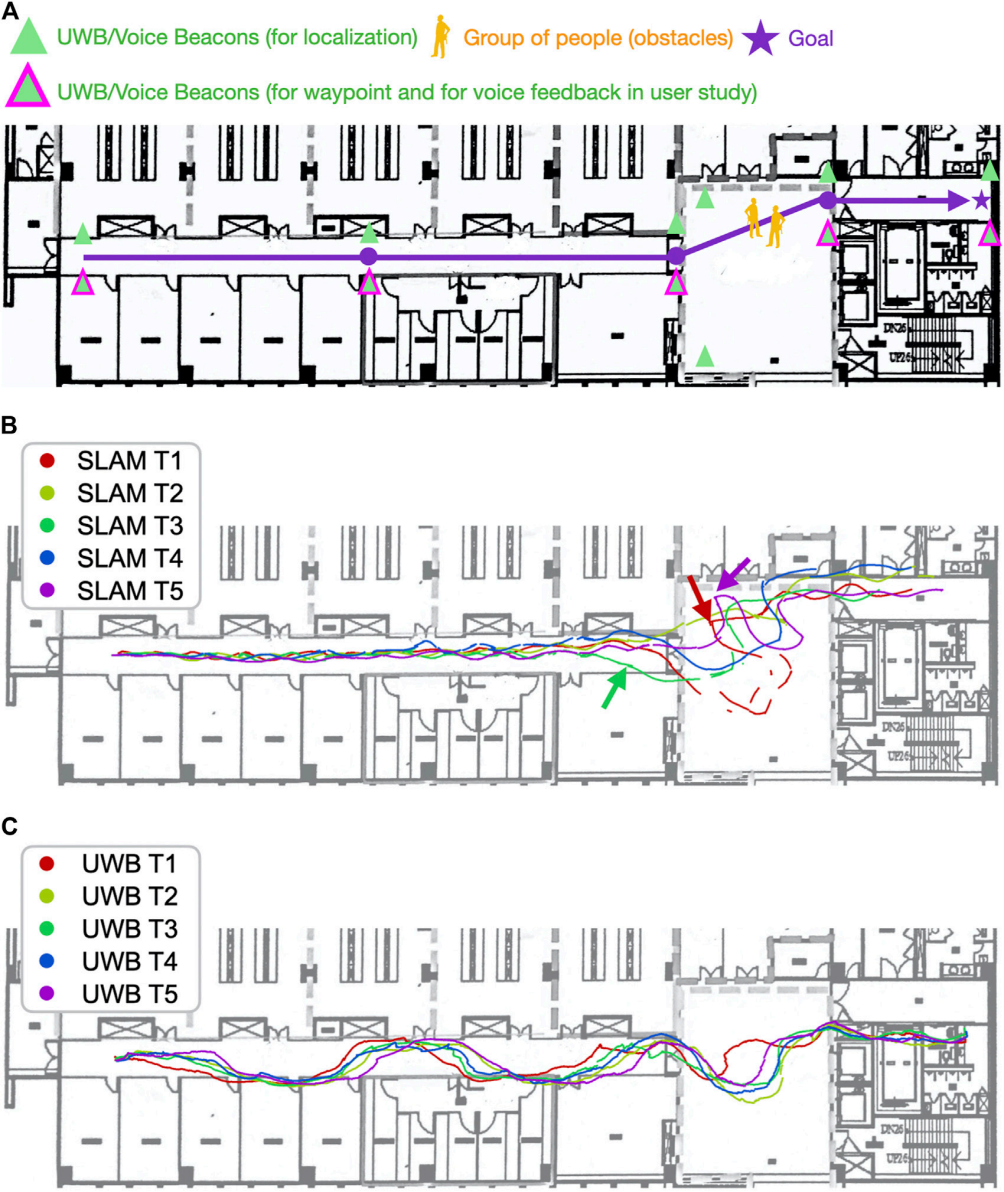
Navigation with the SLAM algorithm in the dynamic environment was less stable and efficient than was navigation with the UWB beacons. **(A)** is the map with the UWB/Voice beacons and the designated route and waypoints. **(B)** visualizes the robot trajectories using proposed DRL with SLAM. **(C)** visualizes the robot trajectories using the proposed DRL with UWB localization. When using the SLAM algorithm, the robot tended to navigate to the right with an unnecessary loop and become trapped periodically (the red and purple arrows). The movement of pedestrians caused the SLAM algorithm to incorrectly estimate the distance traveled in a long corridor. The robot locations estimated by the algorithm (the green arrow) were different from the ground truth. Different results were obtained in the trials conducted with the SLAM algorithm, whereas consistent results were obtained in the trials conducted with the UWB beacons.

A beacon network was deployed in an environment, and the proposed UWB/voice beacons were used. The relative positions of the beacons were be measured. Figure 6A displays the setup of the UWB/voice beacons, and the robot was programmed to follow a certain predefined waypoints. The total setup time, including measuring the relative position by people with distance measure one by one, was approximately 40 min. The dynamic obstacles (i.e., pedestrians), which appear in real-world environments, affected the performance of the SLAM algorithm. These pedestrians were categorized into two types: dynamic obstacles that traveled in the same direction as the robot, which caused perceptual aliasing Lajoie et al. (2019a). The SLAM algorithm would find the features on the dynamic obstacles moving along with the robot and thus was misled that the robot did not move at all. The other type of pedestrian traveled in arbitrary directions, where features on the dynamic obstacle dominated over the ones of the long corridor environment. Two people walked in the environment during the experiment to model the two aforementioned types of dynamic obstacles, shown in Figure 5. Five trials were conducted for each of the methods. All the trials used the same navigation agent, namely a DRL agent, and had the same parameters.

### 5.2 Experimental Results

First, the navigation performance was analyzed. The navigation was more stable and efficient when using UWB positioning than when using the SLAM algorithm for localization. As presented in Table 1, although the end point was successfully reached in all trials, the trial duration when using the SLAM algorithm (317 s) was significantly longer than that when using UWB positioning (217 s; 100 s and 46% less time required than in the method based on the SLAM algorithm). Furthermore, the standard deviation of the trial duration was higher when the SLAM algorithm was used than when UWB positioning was used, implying that the navigation performance was less stable when the SLAM algorithm was used.

**TABLE 1:** Navigation experiment using the baseline methods (SLAM + A*) and proposed methods (UWB + DRL; SLAM + DRL) in static or dynamic environments.

Methods			Duration (s)		Reach G (%)	Human inter Avg. Time/trial	
Localization	Navigation	Env	Mean	SD			Note
SLAM	A*	Static	236.8	12.6	100	2	Baseline
SLAM	A*	Dynamic	—	—	0	5.8	Baseline
UWB	DRL	Dynamic	**217**	12.45	100	0	Proposed 1
SLAM	DRL	Dynamic	317	29.76	100	0	Proposed 2

We found that the baseline method perform well in static environment as the proposed methods, but was unable to reach goal. The proposed one method (UWB + DRL) completed all trials in shortest duration (mean duration marked in bold) without human intervention (risk to collision or trapped until timeout).

The trials performed with the SLAM algorithm (Figure 6B) exhibited various trajectories. The robot periodically tended to navigate to the right in a large loop when the waypoint was in the front at the left side. This navigation result was obtained due to the poor orientation estimation with the aforementioned algorithm, which resulted in the robot wrongly estimating the waypoint to its right rather than its left. This poor orientation estimation also caused the robot to be trapped along the experiment path in the areas indicated by the purple and red arrows in Figure 11b. Although the robot eventually emerged from the trap, it wasted 20–30 s in doing so. Such entrapment might reduce user trust in the robot. The presence of pedestrians also caused the SLAM algorithm to incorrectly estimate the distance traveled in the long corridor. This algorithm falsely estimated that the robot had arrived at entrance of the hall (indicated by the green arrow in Figure 6B); however, the ground truth position of the hall’s entrance was at the intersections between the paths and the white vertical line in Figure 6B. Similar cases of poor estimation in long corridors have been discussed in the study of Song et al. (2019).

Similar navigation performance was observed in the five trials involving UWB positioning (Figure 6C). However, position estimation errors of 1–2 m were observed in the *y* direction in the corridor. This result was obtained because the corridor was excessively narrow, which resulted in the beacon being only 1.74 m away from the robot in the *y* direction. However, the aforementioned phenomenon was not observed when the robot navigated outside the corridor, where the positioning and navigation accuracy increased. A solution for the aforementioned error in the corridor would be to adopt similar distances for the beacons in the *x* direction in *x* direction to have a similar distance as well; however, this measure would considerably increase the number of UWB beacons required.

## 6 User Study for On-handle and On-beacon Feedback

We conducted a user study with eight BVI people [mean age = 44.5 years, standard deviation (SD) = 15.51 years; three women] who had not previously used the proposed system. The detailed demographics of the research participants are presented in Table 2. All the participants were recruited through an association for BVI people in Taiwan. The study protocol was approved by National Chiao Tung University (NCTU-REC-109-088F). Consent was obtained from each participant, and the study procedure and tasks were introduced to them. We expected that the proposed system would be much better than the existing guiding tools for BVI people. Therefore, in the a priori power analysis, the total sample size should be 8 to get 1 as the effect size (ES) under the condition that the alpha is 0.05 and power is 0.8. We carried out the power analysis with one sample *t* test.

**TABLE 2:** Demographic information of our participants.

Id	Gender	Age	Eye sight	Navigation aid
P1	F	23	Blind	Cane
P2	M	52	Blind	Cane
P3	M	36	Blind	Cane
P4	F	30	Blind	Cane
P5	F	46	20/500 for both eyes	Cane
P6	M	64	Blind	Cane
P7	M	70	Blind	Cane
P8	M	35	Blind	Cane

**FIGURE 7:**
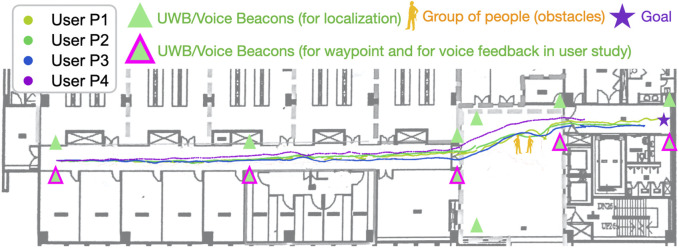
Trajectories for four trials in the user study. The trials were performed with DRL agents. The agents using the same navigation method tended to exhibit a similar navigation performance. In contrast to the A* agent, the DRL agents learned smooth paths to avoid obstacles regardless of the changing environment.

The experimental site was a hallway in the Boai Campus BIO–ICT Building on the campus of National Yang Ming Chiao Tung University (origin is National Chiao Tung University), Taiwan. We selected this location because its environment is quiet; thus, the participants could perceive voice feedback appropriately. The route (see Figure 7) was designed such that the goal point was a restroom, which the BVI community suggested is particularly essential in their daily lives. The route started from an office door and passed through the aforementioned hallway. A total of one to three people were standing along the route, which represents a common scenario encountered in indoor environments. The total length of the route was approximately 65 m, and it contained five UWB beacons.

All the participants were instructed to follow the guiding robot by holding the handle. Each participant was required to complete the route in two runs with the proposed 2 (SLAM + DRL) and the baseline (SLAM + A* path planning) methods. We measured the time taken by each participant to complete each run. All the participants were asked to complete a poststudy questionnaire immediately after each run to understand their 1) perceived difficulty of the route, 2) confidence level when navigating the route, and 3) willingness to use the mobility aid to navigate similar routes. The adopted questionnaire was based on the research of Sauro and Dumas (2009). We also conducted a poststudy interview with the participants after they had completed all the runs and questionnaires. The interviews contained questions on the perceived usefulness of the proposed guiding robot, audio feedback design, handle, and UWB beacons. We excluded data from one participant (P7) because time limitations prevented him from completing the route. However, we still conducted a poststudy interview with him because he had some experience using the proposed system. We finally reported the achieved ES and power after the user study.

### 6.1 Reactions to Interfaces

Table 3 presents an overview of the participants’ performance in navigating the selected route, as well as participants’ responses toward both the haptic interface (i.e., the handle) and the voice feedback. Most of the participants found the designed route easy to navigate. Their perceived difficulty scores were 2.83 and 2.71 out of 7 when using the proposed method and other method, respectively. The participants also perceived a medium level of confidence when using the proposed system (scores of 4.83 and 4.42 out of 7 for the proposed method and other method, respectively). Almost all the participants had a high intention to use the proposed system again on a similar route (scores of 6.00 and 6.14 out of 7 for the proposed method and other method, respectively).

**TABLE 3:** Descriptive Statistics of the answers provided by the participants regarding haptic (i.e., handle) feedback and sound feedback. SD, standard deviation; SE, standard error of the mean; ES, effect size.

No	Questions (on a 7-point scale, 1:strongly disagree to 7:strongly agree)	Mean	SD	Se	ES	Power
Q1	Whether the robot is user friendly?	5.87	1.96	0.26	3.53	1.00
Q2	Whether the speed regulator is useful for you?	3.14	2.29	0.94	0.35	0.22
Q3	Whether the auditory cues provided by the robot is sufficient?	5.25	2.27	0.86	0.51	0.37
	Whether the auditory cues provided by sound-UWB beacons is sufficient?	4.75	2.16	0.82	0.32	0.21
Q4	Whether the handle provided the environment cues in the right time?	5.5	1.41	0.54	0.99	0.81
	Whether the robot provided the auditory cues in the right time?	4.88	1.83	0.69	0.45	0.31
	Whether the sound-UWB beacons provided the auditory cues in the right time?	5	2	0.76	0.47	0.33
Q5	Are there uncomfortable slewing behaviors?	3.12	1.73	0.61	0.51	0.36
	Do you feel dizzy or uncomfortable while being assisted to make turns?	1.5	0.93	0.33	2.7	1.00
	Are there uncomfortable sudden speed changes?	3	2.56	0.91	0.39	0.26

#### 6.1.1 Handle Feedback

In general, the participants found the handle to be convenient to use (average score of 5.87 out of 7; ES = 3.53, Power = 1.00), and did not require considerable effort to learn how to use it. However, they were uncomfortable with the speed of the regulators (average of 3.14 out of 7; ES = 0.35, Power = 0.22). Many participants indicated that the maximum speed of the robot was too slow for them. In addition, the participants believed that the navigation cues were delivered through the handle at the correct time (average score of 5.5 out of 7, ES = 0.99, Power = 0.81).

Overall, our participants found the handle feedback of our system comfortable. The participants felt pretty comfortable when the system gave a turning feedback (an average score of 1.5 out of 7; ES = 2.7, Power = 1.00). Some participants mentioned the unnecessary slewing behavior and sudden speed changes while navigating, however, most still find the robot comfortable with an average score of 3.12 out of 7 (ES = 0.51, Power = 0.36) and average score of 3.00 out of 7 (ES = 0.39, Power = 0.26).

#### 6.1.2 Voice Feedback

Some participants found the auditory cues provided by the on-handle (5.25 out of 7; ES = 0.51, Power = 0.37) and on-beacon (4.75 out of 7; ES = 0.32, Power = 0.21) devices to be informative. Moreover, some of them believed that the information was provided at the correct time (average score 4.88 out of 7; ES = 0.45, Power = 0.31 for the robot; and average score 5.00 out of 7; ES = 0.47, Power = 0.33 for the UWB beacons). Nevertheless, these four responses did not reach significance statistically. In the interviews, two participants (i.e., P2 and P3) suggested that in addition to the location information, detailed environmental information can be provided through the UWB sound beacons. P2 desired additional directional information on the goal, such as the remaining distance to the goal. He also stated that the sudden and sometimes overlapping sound feedback made him startled when navigating. A more pleasing method to provide sound feedback is a potential future research direction. For example, a system can use gentle cues (e.g., gradually louder sound effects) before providing verbal information. Another possible method is to have the user request verbal information rather than passively receive information so that they can expect the sound from the robot and beacons.

### 6.2 Comparison of the Proposed System With Existing Guiding Tools

We compared the proposed system with existing guiding tools (e.g., guide canes and traffic light beeping sounds), and the results are summarized in Table 4. Six out of eight users (75%) preferred the voice feedback in the proposed method; one user (P7) found no difference in the feedback of the various tools; and another user (P2) preferred the beeping sound in existing guide tools. P2 stated that he was startled by the voice feedback from our robot and UWB sound beacons. We believe that a gentler tone of voice feedback can be adopted in the future. The user responses of proposed method were then encoded as one for no difference, two for proposed over existing methods, and 0 otherwise. We found a significant effect toward the proposed method (ES = 2.18, Power = 1.00) In addition, four of the eight participants (50%) preferred the directional information (i.e., information on where the robot was heading) provided by the proposed system (ES = 1.41, Power = 0.97); two participants found no difference between the information provided by existing tools and the proposed method; and two other participants preferred the information provided by existing tools (i.e., their guiding canes). Some of the participants mentioned that the unnecessary slewing behavior of the proposed robot during navigation reduced their preference for the robot. All the participants stated that the proposed system provides more useful environmental information than existing guiding tools do (ES = ∞, Power = 1.00). Seven users preferred both on-handle and on-beacon sound feedback, and one participant preferred only on-handle feedback. Finally, seven of the eight participants preferred using the proposed robotic system for collision avoidance (ES = 2.47, Power = 1.00), and one participant preferred existing guiding tools. We hypothesize that the slow speed and weaving behavior of the proposed robot reduced his preference for it. Future studies can attempt to develop smoother navigation systems that can avoid all possible collisions.

**TABLE 4:** Comparisons of existing and the proposed navigation aid.

No	Questions	Mean	SD	Se	ES	Power
1	Feedback system (sound vs. voice)	1.63	0.74	0.26	2.18	1.00
2	Providing heading info	1.25	0.89	0.31	1.41	0.97
3	Providing POI info (stairs, restroom etc)	2.00	0.00	0.00	∞	1.00
4	Avoid collisions	1.75	0.71	0.25	2.47	1.00

The user responses of proposed method were scored as one for no difference, two for proposed over existing methods, and 0 otherwise. SD: standard deviation; SE: standard error of the mean; ES: effect size.

### 6.3 General Feedback From the Participants

The ability to navigate in unfamiliar environments is crucial for BVI users. Three participants (P4, P5, and P6) mentioned that the proposed system would be particularly useful when navigating in unfamiliar environments, such as department stores, in particular or outdoor areas in general. The proposed navigation system might be less useful in familiar environments because users already have a clear mental map of the surroundings and can orient themselves easily. However, a strong navigation system is still required if users have never visited a location.

Two participants (P2 and P7) stated that the proposed system can be improved if it enables navigation on rough terrain. Although the user study was conducted in an indoor environment, our base robot (i.e., Jackal UGV) can navigate general outdoor terrains, such as roads or grass. Considering user needs, future user studies with the proposed system can be conducted in outdoor environments (e.g., streets).

Two other participants (P3 and P4) suggested that the proposed robot should contain a basket to assist them with daily life. For example, they can place groceries in the basket when shopping. Although such a feature may be irrelevant to the goal of the guiding system (i.e., assisting navigation), the user feedback indicates that an ideal personal guiding robot should be multifunctional. This phenomenon is similar to people expecting other functionalities from smartphones in addition to its main purpose as a communication tool.

### 6.4 Future Improvement According to Participant Feedback

First, the maximum speed and stability of the proposed robot should be increased. All the participants considered the speed adjuster useless because the maximum speed was too low, and they set the robot speed to the highest value. To increase the navigation speed without causing unwanted slewing, a more stable navigation agent is required. The features of stability and speed increase can be added to the reward function of the RL algorithm so that the robot can learn these features.

Second, although the proposed robot system provides more environmental information than existing guiding systems do, the manner in which this information is provided can startle some users. A more appropriate method of providing sound feedback is required. One suggestion is to use a gentler voice to provide information. Another suggestion is to provide information only when requested by the user, which would result in the user expecting to hear voices from the robot.

## 7 Conclusion

In this paper, we propose an assistive navigation system for BVI people. The proposed navigation system includes a DRL-based guiding robot and a handle device with UWB sound beacons. The proposed robot can navigate through routes defined by a series of designated waypoints where UWB beacons are located and use SLAM estimated robot state. The goal points are then fed to the navigation policy, which is trained using a state-of-the-art DRL algorithm. The proposed robot also contains a handle device that can provide a haptic and audio interface that BVI users can interact with. Moreover, the adopted UWB beacons provide environmental information through sound feedback. We conducted a user study in an indoor environment that represents the daily navigation assistance demands of BVI users. The users had confidence in the proposed robot system and a strong intention to use it. Moreover, they considered the proposed system to be user friendly. The auditory cues from the robot and UWB sound beacons were generally sufficient for providing relevant information. The users voiced that the proposed robot system is useful for navigation in unfamiliar environments. They also suggested that the robot have multiple functions and the ability to navigate on rough terrain. Future research directions include increasing the comfort level of the guiding system and using gentler audio cues. Moreover, additional UWB beacons can be used to avoid the effects of dynamic obstacles in environments. A comparison was conducted between the navigation performance achieved when using the SLAM algorithm and UWB positioning for localization. The SLAM algorithm was affected by dynamic obstacles, which resulted in reduced navigation efficiency and stability.

## Data Availability

The raw data supporting the conclusions of this article will be made available by the authors, without undue reservation.
